# Assessment of real-time three-dimensional echocardiography as a tool for evaluating left atrial volume and function in patients with type 2 diabetes mellitus

**DOI:** 10.18632/aging.202218

**Published:** 2020-12-03

**Authors:** Xiuyun Li, Yanyan Dong, Chao Zheng, Pengfei Wang, Maosheng Xu, Chunpeng Zou, Liang Wang

**Affiliations:** 1 Department of Ultrasonic Diagnosis, The Second Affiliated Hospital and Yuying Children’s Hospital of Wenzhou Medical University, Wenzhou 325027, China; 2 Department of Endocrinology, The Second Affiliated Hospital of Zhejiang University School of Medicine, Hangzhou 310000, China

**Keywords:** diabetes mellitus, diabetic nephropathy, real-time, three-dimensional echocardiography

## Abstract

Objective: To assess the value of real-time three-dimensional echocardiography (RT-3DE) in evaluating changes in left atrial volume and function in type 2 diabetes mellitus (DM) and type 2 diabetic nephropathy (DN) patients.

Method: 104 control subjects, 109 DN patients, and 111 DM patients were recruited and underwent RT-3DE. Data pertaining to the left atrium were analyzed using the 3DQA software in order to determine left atrial maximum volume index (LAVImax), left atrial pre-systolic volume index (LAVIp), left atrial minimum volume index (LAVImin), total left atrial ejection fraction (LAEFt), passive left atrial ejection fraction (LAEFp), and active left atrial ejection fraction (LAEFa). Differences between these three groups and correlations between individual index values and E/e' ratios were additionally assessed.

Result: LAVImax, LAVIp, and LAVImin were higher in the DN and DM groups relative to controls, whereas LAEFt and LAEFp were higher in controls relative to DM and DN patients (P < 0.05). LAVImax, LAVIp, and LAVImin in the DN group were significantly higher than those in the DM group, while LAEFt, LAEFp were higher in DM patients relative to DN patients (P < 0.05). The E/e' ratio was also found to be significantly correlated with LAVImax, LAVIp, and LAVImin.

Conclusion: Our results indicate that RT-3DE can be used to assess changes in left atrial volume and function in patients with diabetes and can be used to monitor disease progression-related damage to such left atrial functionality.

## INTRODUCTION

Diabetes mellitus (DM) is a systemic metabolic disease wherein patients suffer from persistently elevated blood glucose levels as a result of impaired insulin production or sensitivity as a result of different environmental and genetic factors. DM patients typically suffer from a number of complications that can reduce the quality of life and lifespan [1–3]. One primary DM complication is diabetic cardiomyopathy (DCM), which was first proposed as a concept in 1972 based on observations of four DM patients suffering from arrhythmia and congestive heart failure despite lacking any symptoms of hypertension, congenital heart disease, or lesions affecting heart valves or coronary arteries [4]. Myocardial disease is now known to occur frequently in DM patients and to be independent of other forms of hypertensive or coronary heart disease.

Abnormal diastolic functionality is an early sign of DCM development [5, 6]. The left atrium serves to connect the left ventricle to the pulmonary veins, and as such its size and functionality are closely associated with left ventricular diastolic function, atrial fibrillation, and related complications [7–9]. Left atrial volume and function are key determinants of diastolic left ventricular filling. As left atrial volume correlates with average left ventricular filling pressure over extended periods of time, it can reliably be used as a stable metric to gauge left ventricular diastolic dysfunction severity and duration. However, traditional echocardiographic approaches are limited in their ability to assess left atrial structure and function. In contrast, real-time three-dimensional echocardiography (RT-3DE) approaches employ full volume imaging and can accurately reflect changes in phase chamber volume during different cardiac cycles [10]. Some studies have shown that patients with hypertension have increased LA volumes and impaired diastolic functions by using RT-3DE [11, 12]. At present, RT-3DE is also used in healthy Children, anemic patients and subclinical hypothyroidism [13–15]. RT-3DE can provide dynamic volume measurement data, which can more accurately assess the volume of the left atrium than traditional methods, as well as provide mechanical parameters. Compared with 2D ultrasound, RT-3DE does not rely on the geometric shape of each section of the left atrium to obtain its overall shape. It can be reliably and easily used to assess left atrial volume changes during different cardiac cycles with greater sensitivity and accuracy than traditional 2D ultrasound analytical approaches [10, 16]. In the present study, we sought to assess whether RT-3DE is an effective means of assessing left atrial volume and function in DM patients.

## RESULTS

### Comparison of baseline participant findings

Patients in the DM and DN groups exhibited significantly higher fasting blood glucose (FBG) and glycated hemoglobin A1C (HbA1c) values relative to control subjects (P = 0.000), whereas these values did not differ significantly between the DM and DN patient groups (P > 0.05). Patients in the DN groups exhibited significantly higher blood urea nitrogen (BUN) and creatinine (CREA) values relative to control groups and DM groups (P = 0.000), whereas these values did not differ significantly between the control groups and DM groups (P > 0.05). No significant differences were detected among the three groups with respect to systolic blood pressure (SBP), diastolic blood pressure (DBP), body mass index (BMI), heart rate (HR), triglycerides (TG), total cholesterol (TC), high density lipoprotein cholesterol (HDL-C), or low-density lipoprotein cholesterol (LDL-C) levels (P > 0.05) (Table 1).

**Table 1:** Comparison of clinical evaluation and biochemical test.

**Variables**	**Group**			***P***
	**Control(n=104)**	**DM(n=111)**	**DN(n=109)**	
AGE(year)	47.93±8.15	50.18±11.62	49.65±12.73	0.302
HR(bpm)	76.88±5.91	75.91±4.98	75.39±5.73	0.144
BMI(kg/m2)	20.91±0.97	20.93±0.92	21.13±0.89	0.146
SBP(mmHg)	117.39±4.80	118.36±4.56	117.25±4.05	0.137
DBP(mmHg)	72.63±3.55	73.31±4.27	72.86±3.70	0.421
FPG(mmol/L)	5.31±0.39	11.42±1.90	11.65±2.18	0.000
HbA1c(%)	5.05±0.42	10.00±1.67	10.21±2.86	0.000
TG(mmol/L)	1.34±0.30	1.33±0.36	1.26±0.37	0.167
TC(mmol/L)	4.42±0.30	4.42±0.34	4.45±0.30	0.673
HDL-C(mmol/L)	1.35±0.18	1.31±0.24	1.30±0.26	0.164
LDL-C(mmol/L)	2.51±0.25	2.47±0.28	2.47±0.29	0.480
BUN(mmol/L)	4.98±1.11	4.91±1.25	10.16±2.84	0.000
CREA(umol/L)	58.96±11.74	59.68±12.10	104.42±44.13	0.000

HR: heart Rate; BMI: body mass Index; SBP: systolic blood pressure; DBP: diastolic blood pressure; FBG: biochemical test includes fasting blood-glucose; HbA1c: glycated hemoglobin A1C; TG: triglycerides; TC: total cholesterol; HDL-C: high density lipoprotein cholesterol; LDL-C: low density lipoprotein cholesterol; BUN: blood urea nitrogen; CREA: creatinine.

### Comparison of conventional echocardiographic parameters

We observed no significant differences in left ventricular ejection fraction (LVEF), left ventricular end-diastolic diameter (LVEDD), left ventricular end-systolic diameter (LVESD), left atrial diameter (LAD), interventricular septal thickness (IVST), left ventricular posterior wall thickness (LVPWT), early diastolic peak velocity at the mitral valve orifice (E), late diastolic peak velocity at the mitral valve orifice (A), or late diastolic peak velocity of the mitral annulus (a') among these three participant groups (P > 0.05), whereas we did find that the average E/A value of patients in the DN group was significantly lower than that of control group patients. In addition, the average E/e' values of DN and DM group patients were significantly higher than those of control group patients, whereas e' and e'/a' values were lower than those of control group patients. Furthermore, the average E/e' value of the DN group patients was significantly elevated relative to that of DM group patients, while the e' and e'/a' values were lower than those of DM group patients (P < 0.05) (Table 2).

**Table 2:** Comparison of conventional echocardiographic parameters.

**Variables**	**Group**			***P***
	**Control(n=104)**	**DM(n=111)**	**DN(n=109)**
LVEDD(mm)	46.73±3.25	46.86±3.78	47.25±2.89	0.497
LVESD(mm)	30.19±2.92	30.58±3.42	30.51±2.80	0.616
LAD(mm)	31.29±2.05	31.52±1.94	31.82±2.36	0.189
IVST(mm)	9.05±0.51	9.02±0.51	9.07±0.52	0.769
PWT(mm)	8.97±0.51	8.94±0.52	8.99±0.53	0.769
EF(%)	64.48±2.24	63.92±2.35	64.06±2.41	0.200
MVE(cm/s)	71.33±5.02	70.50±5.10	70.95±6.04	0.537
MVA(cm/s)	75.63±10.73	76.77±8.22	77.97±6.92	0.150
E/A	0.96±0.16	0.93±0.13	0.91±0.10	0.032
e'(cm/s)	8.95±0.42	7.16±0.52	6.57±0.58	0.000
a'(cm/s)	7.78±0.33	7.90±0.54	7.91±0.68	0.195
e'/a'	1.15±0.05	0.91±0.07	0.83±0.07	0.000
E/e'	7.97±0.59	9.87±0.67	10.70±0.97	0.000

LVEDD: left ventricular end diastolic diameter; LVESD: left ventricular end systolic diameter; LAD: left atrial diameter; IVST: interventricular septum thickness; PWT: posterior wall thickness; EF: ejection fraction; MVE: peak E-wave velocity of the mitral valve; MVA: peak A-wave velocity of the mitral valve; e': early diastolic peak velocity of the septal mitral annulus; a': late diastolic peak velocity of the septal mitral annulus.

### Comparison of RT-3DE parameters

**Figure 1:**
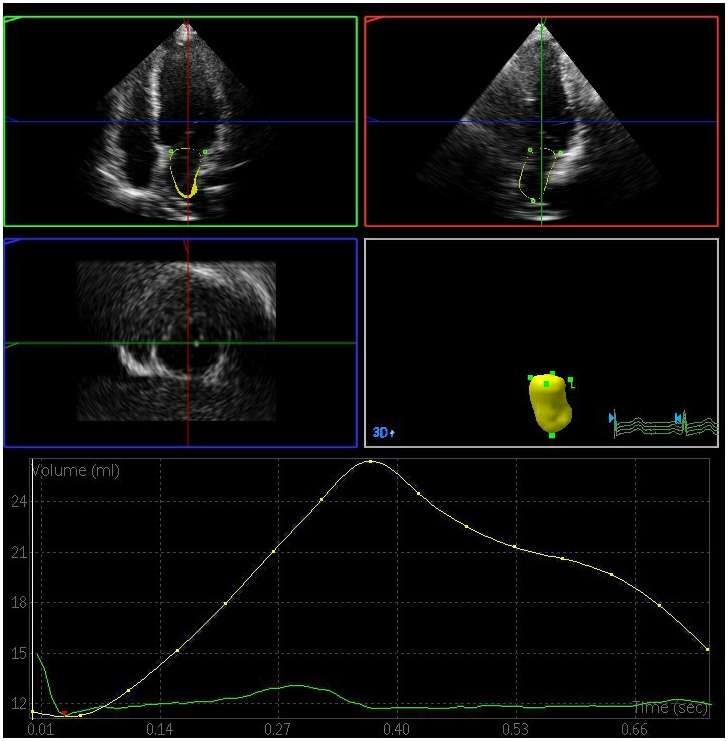
**Left atrial volume quantification by RT-3DE of a case in control group.**

**Figure 2:**
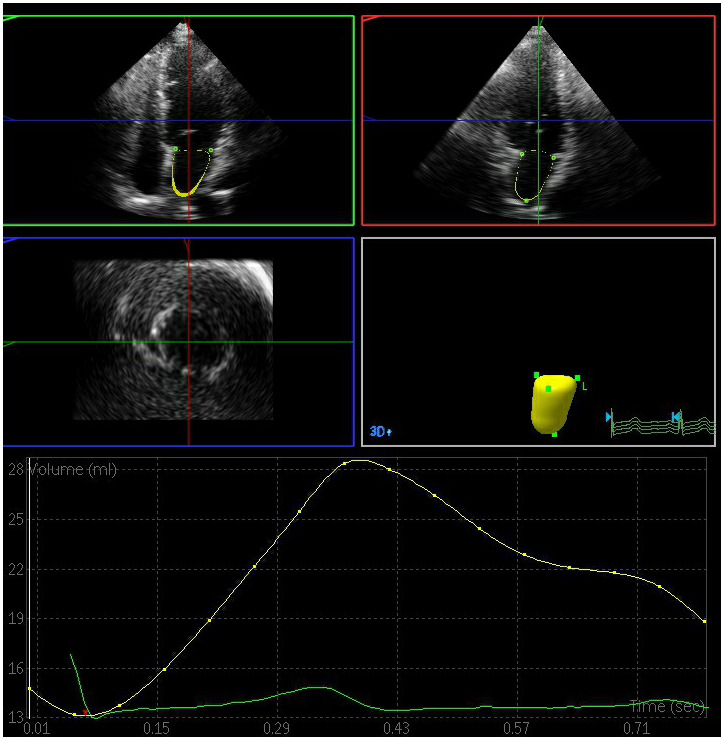
**Left atrial volume quantification by RT-3DE of a case in DM group.**

**Figure 3:**
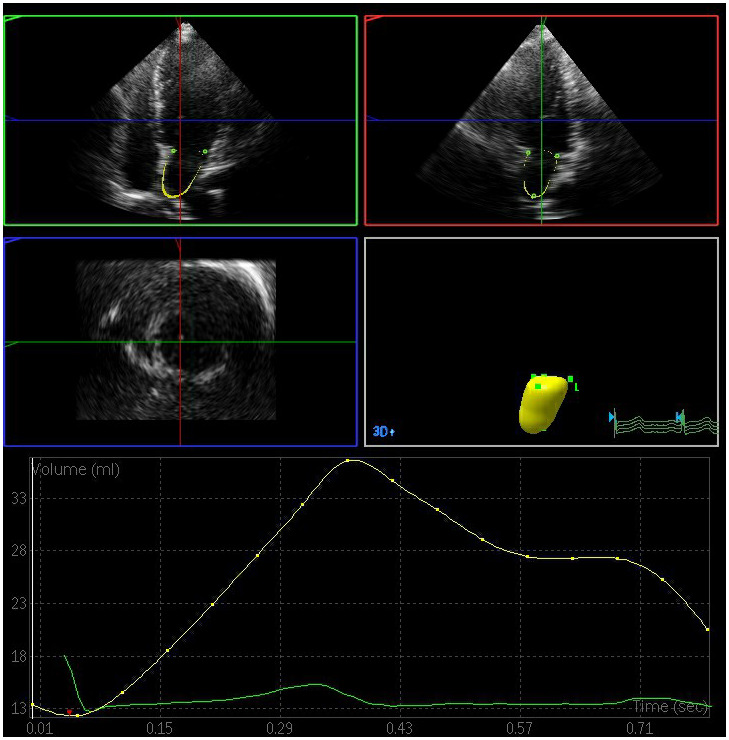
**Left atrial volume quantification by RT-3DE of a case in DN group.**

The left atrial maximum volume (LAVmax), left atrial pre-systolic volume (LAVp), and left atrial minimum volume (LAVmin) were significantly elevated in the DN and DM groups relative to the control group, whereas the total left atrial ejection fraction (LAEFt) and passive left atrial ejection fraction (LAEFp) were significantly lower in the DM and DN groups relative to the control group (P < 0.05) (Figures 1–3). The left atrial maximum volume index (LAVImax), left atrial pre-systolic volume index (LAVIp), and left atrial minimum volume index (LAVImin) were also significantly elevated in the DN group relative to the DM group, whereas the LAEFt and LAEFp parameters were significantly lower in the DN group relative to the DM group (P < 0.05). We also found that active left atrial ejection fraction (LAEFa) was significantly higher in the DN group relative to the DM and control groups (P < 0.05), whereas this parameter did not differ significantly between the DM and control groups (P > 0.05) (Table 3).

**Table 3:** Comparison of parameters of RT-3DE.

**Variables**	**Group**			***P***
	**Control(n=104)**	**DM(n=111)**	**DN(n=109)**	
LAVImax(ml/m2)	21.94±2.06	26.74±3.81	30.94±2.89	0.000
LAVImin(ml/m2)	8.57±1.49	11.44±1.93	13.78±1.68	0.000
LAVIp(ml/m2)	12.74±2.01	17.56±3.32	22.17±2.81	0.000
LAEFt(%)	60.97±5.11	57.29±3.47	55.49±5.11	0.000
LAEFp(%)	42.11±4.81	34.71±4.71	28.87±2.27	0.000
LAEFa(%)	32.40±8.39	34.28±6.63	37.31±7.90	0.000

LAVImax: left atrial maximum volume index; LAVImin: left atrial minimum volume index; LAVIp: left atrial pre-systolic volume index; LAEFt: left atrial total emptying fraction; LAEFp:LA passive emptying fraction; LAEFa: left atrial active emptying fraction.

### Correlation analysis

**Figure 4:**
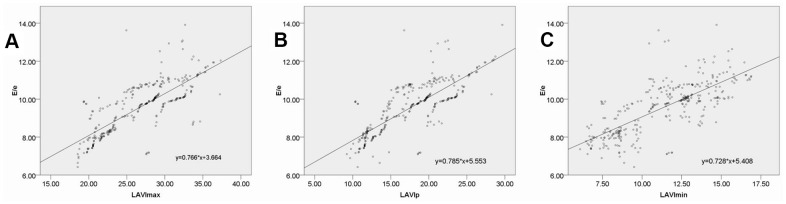
Correlations between the E/e' and the LAVImax (**A**), LAVIp (**B**) and LAVImin (**C**).

E/e' was found to be significantly positively correlated with LAVImax, LAVIp, and LAVImin (r = 0.766, P = 0.000; r = 0.785, P = 0.000; r = 0.728, P = 0.000) (Figure 4).

## DISCUSSION

DM progression is associated with multi-system dysfunction, with cardiovascular diseases being one of the primary complications of progressive DM [17]. DM patients have been shown to be at elevated risk of heart failure even when they do not exhibit traditional risk factors such as hypertension or coronary heart disease [4, 18, 19]. This increased risk of heart failure is believed to be attributable to metabolic changes associated with insulin resistance and hyperglycemia, which can lead to disordered cardiac energy metabolism, cardiac cell hypertrophy, fibrosis, myocardial microcirculation damage, and structural and functional changes within the cardiac tissue. Early pre-symptomatic manifestations of such dysfunction include subclinical left ventricular diastolic dysfunction [5, 6].

In the present study, we found that LAVImax, LAVIp, and LAVImin values in DN and DM patients were higher than those of control patients, with these values being significantly higher in DN patients relative to DM patients (P < 0.05). This suggests that left atrial volume increases in diabetic patients relative to non-diabetic controls, and that this increase is more pronounced in those with DN. We also found that LAEFt and LAEFp values were lower in DM patients relative to controls, and that these values were lower still in DN patients, suggesting that diabetic patients exhibit reductions in left atrial ejection and the conduction functionality relative to non-diabetic controls, and that this functionality is further reduced in those with DN. In addition, our results revealed that the LAEFa was elevated in DN patients relative to DM patients and controls, with no significant difference between these latter two groups. This indicates that left atrial pump functionality is enhanced in those with DN but is unchanged in those with DM relative to non-diabetic controls. This may be because left ventricular diastolic function in DN patients is impacted by both diabetes and by renal complications, further impairing left ventricular compliance and relaxation. This may in turn increase left atrial afterload such that the pump function of the left atrium is increased owing to a consequent increase in atrial contractility that is necessary to maintain left ventricular filling volume.

The E/e' ratio has been validated by the American Society of Echocardiography and the European Society of Cardiovascular Imaging as a reliable index that can be used to evaluate left ventricular diastolic function and that is closely associated with left ventricular filling pressure [20]. In the present study, we found that DM and DN group patients exhibited E/e' ratio values that were significantly higher than those of healthy controls, with these values being higher in DN patients relative to DM patients. This indicates that left ventricular filling pressure progressively increases in DM and DN patients relative to non-diabetic controls. We also detected significant correlations between E/e' and LAVImax, LAVIp, and LAVImin, highlighting the utility of left atrial volume data obtained via RT-3DE as a means of accurately assessing left ventricular filling pressure.

This study has a number of limitations. For one, although we grouped patients according to disease stage, we only included DM and DN patient groups, limiting our ability to resolve disease progression-related changes in RT-3DE findings. In addition, this was a single-center study with a relatively small sample size, limiting the generalizability of our findings. Future large-scale multi-center clinical studies will therefore be needed to validate and expand upon our results.

In conclusion, the progression of diabetes is often associated with progressive increases in left atrial volume that are associated with impaired functionality. Our results emphasize the value of RT-3DE as an effective and efficient approach to accurately evaluating left atrial volume and function in patients with diabetes. This technology may therefore be amenable to widespread clinical application for the diagnosis and treatment of patients with DM at risk of DCM and related complications.

## MATERIALS AND METHODS

The present study received approval from the Research Ethics Committee of the Second Affiliated Hospital of Wenzhou Medical University, and all participants provided written informed consent in accordance with the Declaration of Helsinki.

### Patients

From June 2019 - September 2020, we enrolled 126 newly-diagnosed DM patients and 125 patients newly diagnosed with diabetic nephropathy. The American Diabetes Association diagnostic criteria [21] and the Tervaert criteria [22] were used to diagnose DM and DN patients. Patients were excluded from the present study if they had arrhythmia, congenital cardiovascular disease or if they had a history of cardiovascular disease secondary to hypertension, hyperlipidemia, cardiac dysfunction, or endocrine diseases. The final DM group consisted of 111 patients (58 female, 53 male; mean age 50.18 ± 11.62 years), and the final DN group consisted of 109 patients (56 female, 53 males; mean age 49.65 ± 12.73 years). In addition, we recruited 104 healthy control participants (55 female, 49male; mean age 47.93 ± 8.15 years) for participation in the present study. All participants were free of known cardiovascular risk factors and cardiovascular disease, and were instructed not to consume alcohol or coffee within 24 h prior to examination.

All study participants underwent clinical evaluations to assess weight, height, BMI, blood pressure, medical history, and cardiovascular parameters. In addition, these participants underwent biochemical testing to measure FBG, TG, TC, HDL-C, LDL-C, HbA1c, BUN, and CREA levels.

### Echocardiography

A Philips EPIQ 7C instrument equipped with an x5-1 probe at a frequency of 1-5 MHz was used for the echocardiographic examination of study participants. Initially, conventional 2D and Doppler echocardiography were conducted in order to rule out the possibility that subjects had any previously undetected structural heart diseases including cardiomyopathy, valvular disease, LV hypertrophy, or pericardial disease. A 2D ultrasound approach was used to measure LVEDD, LVESD, LAD, IVST, and LVPWT. The biplane Simpson method was used to calculate the ejection fraction (EF), while early and late diastolic velocity (E and A, respectively) at the mitral valve orifice were determined via spectrum Doppler. Early and late diastolic peak velocity (e' and a', respectively) of the mitral annulus were assessed at the ventricular septum via tissue Doppler. These values were then used to compute E/A, e'/a', and E/e' ratios. Next, the 3D volume probe was utilized. Participants were instructed to hold their breath, after which images were coupled with electrocardiographic recordings. After the apical four-chamber view was clearly displayed, cine-loop clips of four consecutive cardiac cycles were continuously acquired.

### RT-3DE imaging

All the clips were collected at a > 20 frames/s framerate. RT-3DE data were analyzed with the 3DQ advanced analysis software (QLab-Philips v9.1; Philips Medical Systems). The lateral point, anterior point, septal point, inferior point of the mitral annulus, and the left atrial apex in the systolic and diastolic period were manually identified, while the endocardial boundary of the left atrium was identified in each frame via an automated processing method with manual adjustment as necessary. The pulmonary vein mouth and left atrial appendage were excluded so as to yield a 3D model and volume curve of the left atrium such that volume parameters could then be measured by an experienced echocardiologist blinded to subject grouping [12]. Measured volume parameters included: (1) LAVmax at the T wave endpoint, corresponding to the time when the atrial volume was greatest immediately prior to the opening of the mitral valve; (2) LAVmin at the QRS wave endpoint, corresponding to the time when the minimum atrial volume was achieved prior to closure of the mitral valve; and (3) LAVp during the P wave, corresponding to the volume when atrial contraction begins. Left atrial RT-3DE volumes were indexed to the body surface area in order to yield left atrial RT-3DE volume index values (LAVImax, LAVImin, LAVIp). These indices were in turn used to calculate the following left atrial functional parameters: (1) left atrial total emptying fraction (LAEFt) = $\frac{\text{LAVImax}-\text{LAVImin}}{\text{LAVImax}}\times 100\%$, corresponding to atrial reservoir function; (2) left atrial passive emptying fraction (LAEFp) = $\frac{\text{LAVImax}-\text{LAVIp}}{\text{LAVImax}}\times 100\%$, reflecting atrial conduit function; and (3) left atrial active emptying fraction (LAEFa) = $\frac{\text{LAVIp}-\text{LAVImin}}{\text{LAVIp}}\times 100\%$, reflecting atrial pump function.

### Statistical analysis

SPSS v17.0 (SPSS Inc., IL, USA) was used for statistical testing. Data are given as means ± SD, and were compared via LSD tests and one-way ANOVAs, as appropriate. In addition, Pearson correlation analyses were used to evaluate relationships between E/e' and LAVImax, LAVIp, and LAVImin. P < 0.05 was the significance threshold.
